# Lord Haldane's Territorial Hospitals and Nursing Service

**Published:** 1914-08-15

**Authors:** 


### LORD HALDANE'S TERRITORIAL HOSPITALS AND NURSING SERVICE.

| General Hospital. | Situation. | County Association administering the Unit where Local Committees are formed. | Counties included in the area. | Name and Address of Principal Matron. |
|---|---|---|---|---|
| 1st London | London | City of London | — | Miss R. Cox-Davies, Royal Free Hospital, Gray's Inn Road, W.C. |
| 2nd London | Do. | Do. | — | Miss Davies, St. Mary's Hospital, Paddington, W. |
| 3rd London | Do. | London | Middlesex | Miss Barton, Chelsea Infirmary, Chelsea, S.W. |
| 4th London | Do. | Do. | Do. | Miss Ray, King's College Hospital, Lincoln's Inn, W.C. |
| 1st Western | Liverpool | West Lancashire | Cumberland, Westmoreland | Miss Glover, David Lewis Northern Hospital, Liverpool. |
| 2nd Western | Manchester | East Lancashire | — | Miss Sparshott, Royal Infirmary, Manchester. |
| 3rd Western | Cardiff | Glamorgan | All other counties in Wales, Chester, Shropshire(Salop), Hereford and Monmouth | Miss E. M. Wilson, King Edward VII. Hospital, Cardiff. |
| 1st Northern | Newcastle-on-Tyne | Northumberland | Durham | Miss E. F. C. Brown, Royal Victoria Infirmary, Newcastle-on-Tyne. |
| 2nd Northern | Leeds | York (West Riding) | York (North Riding) | Miss E. S. Innes, General Infirmary, Leeds. |
| 3rd Northern | Sheffield | York (West Riding) | York (East Riding) | Miss Smeeton, Royal Infirmary, Sheffield. |
| 4th Northern | Lincoln | Lincoln | Nottingham and Derby | Miss Sheppard, The Hospital, Lincoln. |
| 5th Northern | Leicester | Leicester | Rutland and Stafford | Miss C. E. Vincent, Royal Infirmary, Leicester. |
| 1st Southern | Birmingham | Warwick | Worcester | Miss Buckingham, Queen's Hospital, Birmingham. |
| 2nd Southern | Bristol | Gloucester | Gloucester | Miss Baillie, Royal Infirmary, Bristol. |
| 3rd Southern | Oxford | Oxford | Berks, Buckingham | Miss Watt, Radcliffe Infirmary, Oxford. |
| 4th Southern | Plymouth | Devon | Cornwall, Somerset | Miss Smale, Royal Devon and Exeter Hospital, Exeter. |
| 5th Southern | Gosport | Southampton (Hampshire) | Wilts, Devon | Miss Alcock, Royal Hospital, Portsmouth. |
| 1st Eastern | Cambridge | Cambridge and Isle of Ely | Norfolk, Suffolk, Essex, Northampton, Huntingdon, Bedford, Hertford | Miss Montgomery, Addenbrooke's Hospital, Cambridge. |
| 2nd Eastern | Brighton | Sussex | Surrey, Kent | Vacant. |
| 1st Scottish | Aberdeen | — | The Highlands, including Stirling | Miss Edmondson, Royal Infirmary, Aberdeen. |
| 2nd Scottish | Edinburgh | — | Eastern Counties | Miss Gill, Royal Infirmary, Edinburgh. |
| 3rd Scottish | Glasgow | — | Lanark, Renfrew, Wigtown, Ayr and Kirkcudbright | Miss Gregory Smith, Western Infirmary, Glasgow. |
| 4th Scottish | Glasgow | — | Lanark, Renfrew, Wigtown, Ayr and Kirkcudbright | Miss Melrose, Royal Infirmary, Glasgow. |

NOTE.—The above particulars are taken from "How to Succeed as a Trained Nurse" (Scientific Press, Ltd.), which contains full particulars of the War Service under all Government Departments which a multitude both of men and women are at present anxious to ascertain.

